# Rituximab, Omalizumab, and Dupilumab Treatment Outcomes in Bullous Pemphigoid: A Systematic Review

**DOI:** 10.3389/fimmu.2022.928621

**Published:** 2022-06-13

**Authors:** Peng Cao, Wenjing Xu, Litao Zhang

**Affiliations:** ^1^Graduate school, Tianjin Medical University, Tianjin, China; ^2^Graduate school, Tianjin University of Traditional Chinese Medicine, Tianjin, China; ^3^Department of Dermatology, Tianjin Academy of Traditional Chinese Medicine Affiliated Hospital, Tianjin, China

**Keywords:** rituximab, omalizumab, dupilumab, biologics, bullous pemphigoid

## Abstract

**Background:**

Bullous pemphigoid (BP) is the most common autoimmune subepidermal bullous disease of the skin. First-line treatment of systemic corticosteroids may cause serious adverse events. Rituximab, omalizumab, and dupilumab should be explored as alternative treatment options to improve outcomes.

**Objective:**

To systematically review the rituximab, omalizumab, and dupilumab treatment outcomes in bullous pemphigoid.

**Methods:**

A PubMed, Embase, Web of Science, and Cochrane library search were conducted on March 10, 2022. A total of 75 studies were included using Preferred Reporting Items for Systematic Reviews and Meta-analyses guidelines.

**Results:**

Use of rituximab (n=122), omalizumab (n=53) and dupilumab (n=36) were reported in 211 patients with BP. Rituximab led to complete remission in 70.5% (n=86/122) and partial remission in 23.8% (n=29/122) of patients within 5.7 months, with a recurrence rate of 20.5% (n=25/122). 9.0% (n=11/122) of patients died and infection (6.6%, n=8/122) was the most common adverse event. Omalizumab led to complete remission in 67.9% (n=36/53) and partial remission in 20.8% (n=11/53) of patients within 6.6 months, with a recurrence rate of 5.7% (n=3/53). 1.9% (n=1/53) of patients died and thrombocytopenia (1.9%, n=1/53) was observed as the most common adverse event. Dupilumab led to complete remission in 66.7% (n=24/36) and partial remission in 19.4% (n=7/36) of patients within 4.5 months of treatment without any reported adverse events, with a recurrence rate of 5.6% (n=2/36).

**Conclusions:**

Rituximab, omalizumab, and dupilumab have similar clinical benefits for BP patients. However, rituximab resulted in higher recurrence rates, adverse events, and mortality rates.

**Systematic Review Registration:**

https://www.crd.york.ac.uk/PROSPERO/, identifier CRD42022316454.

## Introduction

Bullous pemphigoid (BP) is the most common autoimmune subepidermal bullous disease of the skin, which mainly affects older adults about 70 years of age (1). The cumulative incidence of BP is estimated as 8.2% per million people, whereas the incidence rate was 34.2% per million people per year (2). The mortality from BP annually in the United States, Europe, and Asia are 11%-23%,13%-41%, and 12-27%, respectively (3). The most common reason for death is opportunistic infections because of long-term iatrogenic immunosuppression (3). BP is characterized by stiff, generally clear blisters, and erythema which are frequently associated with urticarial plaques and almost all patients experience severe pruritus. Typical clinical signs, histology, features on direct or indirect immunofluorescence assays, and positive specific antibodies can all be used to diagnose BP.

The treatment of localized or mild BP is mainly based on topical corticosteroids and can be combined with antibiotics and nicotinamide. Systemic or topical corticosteroids combined with immunosuppressants such as methotrexate, azathioprine, mycophenolate mofetil, cyclophosphamide, or cyclosporine A are used to treat moderate and severe BP. Other treatment modalities include intravenous immunoglobulin, plasma exchange, and immunoadsorption. Long-term application of corticosteroids may cause serious side effects. New therapeutic pharmacologic biologic agents such as rituximab, omalizumab, and dupilumab can selectively inhibit autoantibody formation and inflammatory cascade and it may be a safer and more effective method to treat BP.

Rituximab is a human-mouse chimeric monoclonal antibody against B lymphocyte CD20, consisting of mouse Fab and human FC. Relative molecular mass is about 145000, which can specifically bind to the transmembrane antibody CD20 on the surface of B lymphocytes and clear peripheral B lymphocytes through antibody-dependent cell-mediated cytotoxicity and complement-mediated apoptosis turn, which affects the corresponding antibody production. Rituximab is the earliest and most common treatment for bullous dermatoses, which has been approved by the Food and Drug Administration (FDA) for treating Pemphigus vulgaris. The European Academy of Dermatology and Venereology (EADV) currently suggests it as a third-line treatment for BP (4).

Omalizumab is a recombinant humanized anti-immunoglobulin E monoclonal antibody. It blocks the binding of IgE to FcϵRI and FcϵRII on the surface of mast cells, basophils, and dendritic cells by specifically binding to the free IgE Cϵ3 region to reduce inflammatory cell activation and inflammatory cascade (5, 6). Studies have shown that IgE is involved in BP pathogenesis. IgE deposition was observed in the epidermal basement membrane zone in 41% of BP patients (7), and there is a higher Bullous Pemphigoid Disease Area Index (BPDAI) score in patients with BP with IgE deposits along the dermal-epidermal junction than in patients without linear IgE deposits (8). The level of Anti-BP180 IgE correlates with BP pathogenesis and BP activity. However, due to varying assays, the fraction of BP patients with combined anti-BP180 IgE autoantibodies remains unknown, with anti-BP180 IgE autoantibody-positive rates ranging from 22% to 100% (9).

Dupilumab is a humanized IgG4 monoclonal antibody that targets the interleukin (IL)-4 receptor alpha chain. Dupilumab can inhibit T helper (Th) 2 cell differentiation, the transformation of Treg cells into ex-Treg cells in the context of allergic inflammation, and IgE production by B cells, driven by T follicular helper-derived IL-4. It can also prevent IL-4-related vascular endothelium dysfunction and inhibit ILC2 induction *via* eosinophils and basophils (10). The pathogenesis of BP involves the Th1/Th2 inflammatory reaction and the expression of the cytokine IL-4 is increased in the skin, serum, and herpes fluid of BP patients (11).

Therefore, this comprehensive study aims to systematically assess and evaluate the available reports on rituximab, omalizumab, and dupilumab and their outcomes in BP patients.

## Methods

### Search Strategy and Eligibility

The systematic review was performed following the Preferred Reporting Items for Systematic Reviews and Meta-Analyses (PRISMA) guidelines (12) and registered with the PROSPERO international prospective registry (CRD42022316454). A comprehensive review was performed on articles published from inception to March 10, 2022, in the following databases: PubMed, Embase, Web of Science, and Cochrane library. The following search string combining Medical Subject Headings (MeSH) terms and related words: ((Pemphigoid, Bullous) OR (Bullous Pemphigoid) OR (Pemphigoid) OR (Pemphigoids)) AND ((Rituximab) OR (CD20 Antibody, Rituximab) OR (Rituximab CD20 Antibody) OR (Mabthera) OR (IDEC-C2B8 Antibody) OR (IDEC C2B8 Antibody) OR (IDEC-C2B8) OR (IDEC C2B8) OR (GP2013) OR (Rituxan)) were searched. Vocabulary and syntax were adapted to be appropriate for each database. Additionally, the reviewers manually searched the references of the articles independently to identify any additional articles that search engines may have otherwise missed. A similar strategy was used in the search for omalizumab and dupilumab publications.

Studies were included that documented patients diagnosed with BP and reported resolved outcomes for the treatment of BP with rituximab, omalizumab, and dupilumab. The diagnosis of BP needs to meet at least one of the followings:(1) Histopathology reveals subepidermal blistering with eosinophilic infiltration. (2) Direct immunofluorescence: basement membrane containing IgG, IgM, and C3 deposits. (3) Indirect immunofluorescence:the presence of anti-basement band antibody IgG in the patient’s serum. (4) Positivity for BP180 and/or BP230 in an enzyme-linked immunosorbent assay. Studies were excluded if they did not report any efficacy data and that documented patients diagnosed with drug-induced bullous pemphigoid. There will not be any language or geographic restrictions.

### Outcomes

1. Resolution outcomes on biologic treatment:
1) Complete remission: The total resolution of BP lesions. The publications used the terms “complete remission”, “complete response”, “complete control”, and “symptom-free”.
2) Partial remission: Improvement yet lack the complete resolution of BP lesions. The publications used the terms “partial remission,” “partial response”, “improved”, and “clinical improvement”.
3) No remission: no changes in BP lesions. The publications used the terms “no resolution” or “no response”.
4) Deterioration: exacerbation of BP lesions.
2. Time to remission:
The duration between biologic treatment starting and reports of resolution outcomes.
3. Recurrence:
Recurrence of BP lesions during biologic treatment or after biologic treatment stopped. The publications used the terms “new blister” or “recurrence of bullae”.
4. Adverse events.

### Study Selection and Data Extraction

Two reviewers (Cao and Xu) independently screened all the studies identified by the search strategy, screen titles, and abstracts, followed by the full text of potentially eligible studies. Any disagreement was resolved through discussion with a third reviewer (Zhang).

For each selected study, the following information was extracted into an electronic form: first author, publication year, study design, number of patients, sex, age, BP duration, follow-up period, detected antibodies (anti-BP180 IgG, anti-BP230 IgG, anti-BP180 IgE, anti-BP230 IgE, elevated total IgE), eosinophilia, previous treatment, concomitant treatment, resolution outcomes (complete remission, partial remission, no remission, deterioration), time to remission, recurrence, and adverse events.

### Quality Assessment

The Oxford Centre for Evidence-Based Medicine 2009 Levels of Evidence was used to evaluate the quality of evidence.

### Statistical Analyses

Data were analyzed using descriptive statistics. Categorical variables are presented as numbers and percent and continuous variables as mean and range.

## Results

**Figure 1:**
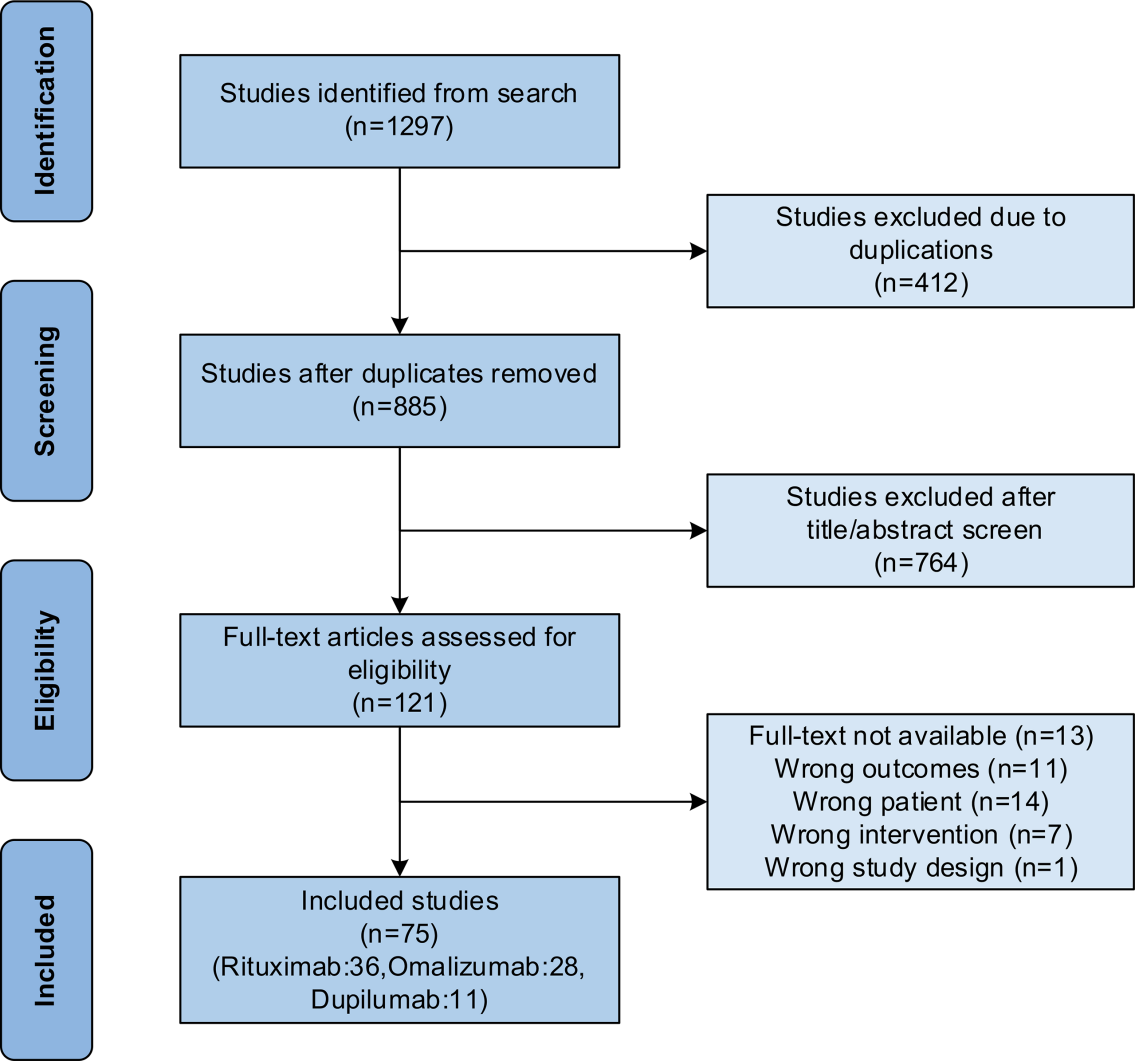
Flow chart of study selection following the Preferred Reporting Items for Systematic Reviews and Meta-Analyses (PRISMA) guidelines.

As shown in **Figure 1**, a total of 75 publications were included for analysis as 412 duplicated publications were excluded, 764 were excluded after reading titles and abstracts, and 46 were excluded after further reading from the 1,297 retrieved publications. The rituximab group had 36 publications (13–48), including 5 prospective studies, 9 retrospective studies, and 22 case series or reports. The omalizumab group had 28 publications (17, 49–75), including 1 prospective study, 1 retrospective study, and 26 case series or reports. While the dupilumab group had 11 publications (59, 73, 76–84), including 2 retrospective studies and 9 case series or reports.

A total of 211 patients were included, of whom 122 received rituximab, 53 received omalizumab, and 36 received dupilumab (**Table 1**). Among these 211 patients (mean age were 68.0 years old, ranging from 0.3 to 95.0 years old), 43.6% (n=92/211) were women, 42.2% (n=89/211) were men, and 14.2% (n=30/211) had no gender information reported.

**Table 1:** Summary of demographic information in patients with BP.

Demographics	Rituximab	Omalizumab	Dupilumab	Total
Patients, n (%)	122 (100.0)	53 (100.0)	36 (100.0)	211 (100.0)
Sex, n (%)				
Female	53 (43.4)	24 (45.3)	15 (41.7)	92 (43.6)
Male	45 (36.9)	26 (49.1)	18 (50.0)	89 (42.2)
NR	24 (19.7)	3 (5.7)	3 (8.3)	30 (14.2)
Age, y				
Mean	65.9	68.2	74.3	68.0
Range	0.3-88	0.4-95.0	22.0-91.0	0.3-95.0
NR, n (%)	15 (12.3)	2 (3.8)	3 (8.3)	20 (9.5)
BP duration, months				
Mean	25.4	9.6	19.2	19.3
Range	1.0-92.0	1.0-48.0	1.0-240.0	1.0-240.0
NR, n (%)	59 (48.4)	14 (26.4)	3 (8.3)	76 (36.0)
Follow-up period, months				
Mean	21.9	5.6	8.6	19.1
Range	1.0-38.0	2.0-10.0	5.0-12.0	1.0-38.0
NR, n (%)	23 (18.9)	36 (67.9)	31 (86.1)	90 (42.7)
Detected antibodies, n (%)				
Anti-BP180 IgG +	80 (65.6)	25 (47.2)	3 (8.3)	108 (51.2)
Anti-BP230 IgG +	59 (48.4)	14 (26.4)	1 (2.8)	74 (35.1)
NR, n (%)	40 (32.8)	26 (49.1)	33 (91.7)	99 (46.9)
Anti-BP180 IgE +	N/A	4 (7.5)	0 (0.0)	4 (1.9)
Anti-BP230 IgE +	N/A	3 (5.7)	0 (0.0)	3 (1.4)
Elevated total IgE	N/A	34 (64.2)	6 (16.7)	40 (19.0)
Eosinophilia, n (%)	N/A	18 (34.0)	4 (11.1)	22 (10.4)
Previous treatment for BP, n (%)				
Corticosteroids	67 (54.9)	51 (96.2)	24 (66.7)	142 (67.3)
Immunosuppressants				
Methotrexate	13 (10.7)	5 (9.4)	8 (22.2)	26 (12.3)
Mycophenolate mofetil	22 (18.0)	7 (13.2)	5 (13.9)	34 (16.1)
Azathioprine	14 (11.5)	9 (17.0)	2 (5.6)	25 (11.8)
Cyclosporine	5 (4.1)	1 (1.9)	1 (2.8)	7 (3.3)
Cyclophosphamide	6 (4.9)	2 (3.8)	1 (2.8)	9 (4.7)
Tacrolimus	1 (0.8)	0 (0.0)	0 (0.0)	1 (0.5)
Antibiotics				
Dapsone	22 (18.0)	7 (13.2)	1 (2.8)	30 (14.2)
Doxycycline	2 (1.6)	11 (20.8)	6 (16.7)	19 (9.0)
Minocycline	4 (3.3)	2 (3.8)	0 (0.0)	6 (2.8)
Tetracycline	0 (0.0)	3 (5.7)	0 (0.0)	3 (1.4)
Azithromycin	0 (0.0)	1 (1.9)	0 (0.0)	1 (0.5)
Nicotinamide	4 (3.3)	7 (13.2)	5 (13.9)	16 (7.6)
Intravenous immunoglobulin	15 (12.3)	11 (20.8)	5 (13.9)	31 (14.7)
Immunoadsorption	1 (0.8)	0 (0.0)	0 (0.0)	1 (0.5)
Plasma exchange	1 (0.8)	0 (0.0)	0 (0.0)	1 (0.5)
Antihistamines	2 (1.6)	5 (9.4)	1 (2.8)	8 (3.8)
Rituximab	N/A	6 (11.3)	2 (5.6)	8 (3.8)
Omalizumab	1 (0.8)	N/A	5 (13.9)	6 (2.8)
None	1 (0.8)	0 (0.0)	0 (0.0)	1 (0.5)
NR	47 (38.5)	0 (0.0)	8 (22.2)	55 (26.1)
Concomitant treatment, n (%)				
Corticosteroids	86 (70.5)	36 (67.9)	23 (63.9)	145 (68.7)
Immunosuppressants				
Azathioprine	4 (3.3)	3 (5.7)	9 (25.0)	16 (7.6)
Methotrexate	4 (3.3)	0 (0.0)	3 (8.3)	7 (3.3)
Mycophenolate mofetil	10 (8.2)	2 (3.8)	2 (5.6)	14 (6.6)
Cyclophosphamide	1 (0.8)	0 (0.0)	1 (2.8)	2 (0.9)
Cyclosporine	1 (0.8)	0 (0.0)	0 (0.0)	1 (0.5)
Antibiotics				
Dapsone	6 (4.9)	1 (1.9)	0 (0.0)	7 (3.3)
Doxycycline	3 (2.5)	7 (13.2)	2 (5.6)	12 (5.7)
Tetracycline	0 (0.0)	1 (1.9)	0 (0.0)	1 (0.5)
Nicotinamide	0 (0.0)	2 (3.8)	1 (2.8)	3 (1.4)
Intravenous immunoglobulin	17 (13.9)	1 (1.9)	0 (0.0)	18 (8.5)
Plasma exchange	1 (0.8)	0 (0.0)	0 (0.0)	1 (0.5)
Immunoadsorption	3 (2.5)	0 (0.0)	0 (0.0)	3 (1.4)
Antihistamines	0 (0.0)	6 (11.3)	0 (0.0)	6 (2.8)
Daclizumab	1 (0.8)	0 (0.0)	0 (0.0)	1 (0.5)
Omalizumab	0 (0.0)	N/A	1 (2.8)	1 (0.5)
None	14 (11.5)	8 (15.1)	3 (8.3)	25 (11.8)
NR	40 (32.8)	5 (9.4)	1 (2.8)	46 (21.8)

BP, bullous pemphigoid; NR, not reported; N/A, not applicable.

### Rituximab

The average course of the disease was 25.4 months (ranging from 1.0 to 92.0 months). No IgG antibody information was reported for 32.8% (n=40/122) of patients in the rituximab groups, and 65.6% (n=80/122) presented anti-BP180 IgG positive and 48.4% (n=59/122) were anti-BP230 IgG positive.

Before rituximab treatment, 54.9% (n=67/122) received corticosteroids but failed to be treated; 10.7% (n=13/122), 18.0% (n=22/122), 11.5% (n=14/122), 4.1% (n=5/122), 4.9% (n=6/122), 0.8% (n=1/122) received immunosuppressants methotrexate, mycophenolate mofetil, azathioprine, cyclosporine, cyclophosphamide, and tacrolimus, respectively, but failed; 18.0% (n=22/122), 1.6% (n=2/122), 3.3% (n=4/122) received antibiotics dapsone, doxycycline and minocycline, respectively, but failed; 3.3% (n=4/122), 12.3% (n=15/122), 0.8% (n=1/122), 0.8% (n=1/122), 1.6% (n=2/122) received nicotinamide, intravenous immunoglobulin, immunoadsorption, plasma exchange, antihistamines, respectively, but failed; 0.8% (n=1/122) received omalizumab but failed, and 0.8% (n=1/122) did not receive any treatment.

After rituximab treatment, 70.5% (n=86/122) of patients had complete remission, 23.8% (n=29/122) had partial remission, 4.9% (n=6/122) showed no remission, and 0.8% (n=1/122) had deteriorated (**Table 2**). The average time to remission was 5.7 months (range, 1.0-13.0 months). The mean follow-up time after treatment was 21.9 months (range, 1.0-38.0 months). Also, 20.5% (n=25/122) patients recurred, 70.5% (n=86/122) did not recur, and 3.3% (n=4/122) did not report.

**Table 2:** Summary of rituximab, omalizumab, and dupilumab treatment outcomes in patients with BP.

Treatment outcomes	Rituximab	Omalizumab	Dupilumab
Patients, n (%)	122 (100.0)	53 (100.0)	36 (100.0)
Resolution outcomes, n (%)			
Complete remission	86 (70.5)	36 (67.9)	24 (66.7)
Partial remission	29 (23.8)	11 (20.8)	7 (19.4)
No remission	6 (4.9)	6 (11.3)	5 (13.9)
Deterioration	1 (0.8)	0 (0.0)	0 (0.00)
Time to remission, months			
Mean	5.7	6.6	4.5
Range	1.0-13.0	0.5-15.0	1.0-15.0
NR, n (%)	48 (39.3)	17 (32.1)	11 (30.6)
BP recurrence, n (%)			
Yes	25 (20.5)	3 (5.7)	2 (5.6)
No	86 (70.5)	42 (79.2)	26 (72.2)
NR	4 (3.3)	2 (3.8)	3 (8.3)
Adverse events, n (%)			
None	73 (59.8)	34 (64.2)	30 (83.3)
Death	11 (9.0)	1 (1.9)	0 (0.0)
Infection	8 (6.6)	0 (0.0)	0 (0.0)
Altered mental status	4 (3.3)	0 (0.0)	0 (0.0)
Anemia	2 (1.6)	0 (0.0)	0 (0.0)
Tachycardia	1 (0.8)	0 (0.0)	0 (0.0)
Compression fracture	1 (0.8)	0 (0.0)	0 (0.0)
Prostate cancer	1 (0.8)	0 (0.0)	0 (0.0)
Metastatic breast cancer	1 (0.8)	0 (0.0)	0 (0.0)
Mucoepidermoid carcinoma	1 (0.8)	0 (0.0)	0 (0.0)
Dyspnea	1 (0.8)	0 (0.0)	0 (0.0)
Thrombocytopenia	0 (0.0)	1 (1.9)	0 (0.0)
NR	19 (15.6)	17 (32.1)	6 (16.7)

BP, bullous pemphigoid; NR, not reported; N/A, not applicable.

No adverse events occurred in 59.8% (n=73/122) of patients, information related to adverse events was not reported in 15.6% (n=19/122), and 9.0% (n=11/122) of patients died. The most common adverse event was infection (6.6%, n=8/122), followed by altered mental status (3.3%, n=4/122), anemia (1.6%, n=2/122), tachycardia (0.8%, n=1/122), compression fracture (0.8%, n=1/122), prostate cancer (0.8%, n=1/122), metastatic breast cancer (0.8%, n=1/122), mucoepidermoid carcinoma (0.8%, n=1/122) and dyspnea (0.8%, n=1/122).

### Omalizumab

Among all patients, the average course of disease was 9.6 months (ranging from 1.0 to 48.0 months). No IgG antibody information was reported for 49.1% (n=26/53) of patients in the omalizumab groups, and 47.2% (n=25/53) presented anti-BP180 IgG positive and 26.4% (n=14/53) were anti-BP230 IgG positive, while 7.5% (n=4/53) presented anti-BP180 IgE positive and 5.7% (n=3/53) were anti-BP230 IgE positive. Also, 64.2% (n=34/53) of patients showed an apparent increase in total IgE levels and eosinophils levels of 34.0% (n=18/53).

Before omalizumab treatment, 96.2% (n=51/53) received corticosteroids but failed; 9.4% (n=5/53), 13.2% (n=7/53), 17.0% (n=9/53), 1.9% (n=1/53), 3.8% (n=2/53) received immunosuppressants methotrexate, mycophenolate mofetil, azathioprine, cyclosporine, and cyclophosphamide, respectively, but failed; 13.2% (n=7/53), 20.8% (n=11/53), 3.8% (n=2/53), 5.7% (n=3/53), 1.9% (n=1/53) received antibiotics dapsone, doxycycline, minocycline, tetracycline, and azithromycin, respectively, but failed; 13.2% (n=7/53), 20.8% (n=11/53), 9.4% (n=5/53) received nicotinamide, intravenous immunoglobulin, and antihistamines, respectively, but failed; and 11.3% (n=6/53) received rituximab but failed.

After omalizumab treatment, 67.9% (n=36/53) of patients had complete remission, 20.8% (n=11/53) had partial remission, 11.3% (n=6/53) showed no remission, and no patients had deteriorated. The average time to remission was 6.6 months (range, 0.5-15.0 months). The mean follow-up time after treatment was 5.6 months (range, 2.0-10.0 months), 5.7% (n=3/53) patients recurred, 79.2% (n=42/53) did not recur, and 3.8% (n=2/53) did not report.

No adverse events occurred in 64.2% (n=34/53) of patients, information related to adverse events was not reported in 32.1% (n=17/53), and 1.9% (n=1/53) of patients died. The most common adverse event was thrombocytopenia (1.9%, n=1/53).

### Dupilumab

Among all patients, the average course of the disease was 19.2 months (ranging from 1.0 to 240.0 months). No IgG antibody information was reported for 91.7% (n=33/36) of patients in the dupiluma groups, 8.3% (n=3/36) presented anti-BP180 IgG positive, and 2.8% (n=1/36) were anti-BP230 IgG positive. Moreover, 16.7% (n=6/36) appeared to increase in total IgE level and eosinophils level of 11.1% (n=4/36) patients raised.

Before dupilumab treatment, 66.7% (n=24/36) received corticosteroids but failed; 22.2% (n=8/36), 13.9% (n=5/36), 5.6% (n=2/36), 2.8% (n=1/36), 2.8% (n=1/36) received immunosuppressants methotrexate, mycophenolate mofetil, azathioprine, cyclosporine, and cyclophosphamide, respectively, but failed; 2.8% (n=1/36), 16.7% (n=6/36) received antibiotics dapsone and doxycycline respectively but failed; 13.9% (n=5/36), 13.9% (n=5/36), 2.8% (n=1/36) received nicotinamide, intravenous immunoglobulin, and antihistamines, respectively, but failed; 5.6% (n=2/36), 13.9% (n=5/36) received rituximab and omalizumab, respectively, but failed.

After dupilumab treatment, 66.7% (n=24/36) of patients had complete remission, 19.4% (n=7/36) had partial remission, 13.9% (n=5/36) showed no remission, and no patients had deteriorated. The average time to remission was 4.5 months (range, 1.0-15.0 months). The mean follow-up time after treatment was 8.6 months (range, 5.0-12.0 months), 5.6% (n=2/36) patients recurred, 72.2% (n=26/36) did not recur, and 8.3% (n=3/36) did not report.

No adverse events occurred in 83.3% (n=30/36) of patients, information related to adverse events was not reported in 16.7% (n=6/36), and no patients died.

## Discussion

A total of 211 patients were included in this systematic review and the ratio of men to women was 0.97/1. There was no gender difference and the average age was 68.0, which was lower than the previously reported age of BP at the end of 70 years (1). This may be since the number of patients included in this systematic review was less but in comparison, it may be due to the tendency of BP patients to seek medical treatment and agree to receive biological agents decreasing with age. Whether there is a younger trend in the incidence of BP needs a broader epidemiological investigation.

Rituximab, omalizumab, and dupilumab have similar clinical benefits in treating BP. The complete remission ratio was 70.5%, 67.9% and 66.7%, respectively, and the partial remission ratio was 23.8%, 20.8% and 19.4%, respectively. One patient received complete remission by rituximab after the failure of omalizumab, five patients received complete remission by dupilumab after the failure of omalizumab, and one patient received partial remission by dupilumab after the failure of rituximab. This indicates the intricacies of the types of autoantibodies, their specific pathogenic actions, and the wide inherent inter-individual variations in BP (85). One patient received both omalizumab and dupilumab and achieved complete remission after 3 months of treatment and no recurrence was found after 10 months of follow-up. Whether treatment with two or more new biological agents simultaneously can produce more clinical benefits needs to be further explored.

Some individuals with refractory BP were resistant to treatment or had a high recurrence rate. In this group of patients, standard corticosteroids and immunosuppressants were ineffective. Some patients with refractory BP have benefited from the clinical use of rituximab, omalizumab, and dupilumab. Of the 211 patients included in the systematic review, a large proportion received biologics after failing treatment with corticosteroids, immunosuppressants, and antibiotics. It achieved complete or partial remission, and rituximab, omalizumab, and dupilumab could be the safe and effective alternative therapy for refractory BP. Only one patient, who had not received any treatment before receiving the biologic, achieved complete remission and no recurrence after rituximab treatment suggests that patients can benefit from the direct use of biologics in the early stage of the disease.

The exact mechanism by which rituximab, omalizumab, and dupilumab produce clinical remission in BP is currently unclear. Rituximab against the CD20 surface protein expressed on B-cell lymphocytes which produce the autoantibodies involved in BP pathogenesis. Once bound, the Fc portion of the antibody recruits immune effector cells for lysis of such antibody-producing B cells, leading to BP remission (86). Protected and non-pathogenic B cells were generated during repopulation when rituximab cleared pathogenic B cells that might contribute to long-term clinical remission (87).

IgE autoantibodies were involved in BP pathogenesis. The binding of Anti-BP180 IgE to FcϵRI on the surface of mast cells and eosinophils promotes inflammatory cell activation and inflammatory cascade response, thus leading to tissue damage and blister formation. In contrast, the binding of Anti-BP180 IgE to the extracellular region of BP180 might lead to internalization of BP180 and blister formation (74, 88, 89). Omalizumab leads to BP remission by directly blocking the process of IgE binding to cell surface FcϵRI. In addition, serum total IgE levels were elevated in 64.2% (n=34/53) of patients in the omalizumab group. Elevated serum total IgE levels correlate with the severity of BP, and omalizumab isolates free IgE, and prevents its binding to the IgE receptor FcϵRI, thus helping such BP patients to achieve clinical remission (89, 90).

Complex immune interactions associated with the Th2 axis, including the cytokines IL-4 and IL-13 and chemokines and eosinophils, are involved in the pathogenesis of BP. The IL-4 receptor alpha antagonist dupilumab inhibits B-cell proliferation, eosinophil chemotaxis, and Th2 chemokines expression by blocking IL-4 and IL-13 signaling and ultimately leads to BP remission (91).

The recurrence rate in the rituximab group was higher than that in omalizumab and dupilumab groups (20.5% vs. 5.7% vs. 5.6%). This may be due to the longer duration of BP in the rituximab group (25.4 vs. 9.6 vs. 19.2 months). One study found that the recurrence rate increased in patients with a longer duration of disease who received rituximab and the remission rate decreased (92). Another study found that patients with pemphigus who received rituximab after a long course of the disease and after conventional treatment failed, had a higher recurrence rate than those who received rituximab directly at the early stage of the disease (93). The later introduction of rituximab means more pathogenic B cell clones. The failure of rituximab treatment and the recurrence of patients after treatment are associated with the prolongation of the course of disease caused by conventional treatment (94).

Furthermore, longer follow-up time in the rituximab group was also associated with a higher recurrence rate (21.9 vs. 5.6 vs. 8.6 months). Relapse of BP generally occurs 12-18 months after treatment, which means that the rituximab group with a follow-up of 21.9 months was able to assess recurrence rates accurately. In contrast, the omalizumab and dupilumab groups may have had a lower recurrence rate than the actual value because of the short follow-up. Mechanistically, relapse is associated with a higher percentage of memory B cells, a decreased proportion of naïve and/or transitional B cells, or lower peak serum levels of B-cell activating factors (13). Besides, *in vitro* studies confirmed that rituximab could not clear the IgA-secreting plasma cells (95), which is one of the reasons for the high recurrence rate in the rituximab group.

The incidence of no adverse events in the dupilumab group was higher than in the omalizumab and rituximab groups. In contrast, the incidence of no adverse events in the omalizumab group was similar to that in the rituximab group. Noa Kremer et al. reported that adverse events in BP treated with omalizumab and rituximab were similar at 20% and 24%, respectively (96).

The main adverse event of rituximab treatment of BP is infection (6.6%, n=8/122), which is related to rituximab’s mechanism in clearing B lymphocytes. Rituximab should not be used in patients with active infection and impaired immune function. Infection caused by rituximab treatment of BP is generally mild. However, older patients and patients treated with large doses of corticosteroids or immunosuppressants should guard against the emergence of severe infection (97). The main adverse event of omalizumab in the treatment of BP is thrombocytopenia (1.9%, n=1/53). In a report on the treatment of asthma, the main adverse event related to omalizumab is anemia, with an incidence of 1-2/1000 (98). No adverse events have been reported in the treatment of BP with dupilumab. In treating atopic dermatitis, the most common side effect of dupilumab is injection site reaction, which mainly consists of transient erythema or edema (99) and the only specific side effect is conjunctivitis (100).

There was no death in the dupilumab group. One patient in the omalizumab group died of pneumonia. Two patients in the rituximab group died of acute respiratory failure. Two patients died of heart failure, two patients died of severe pneumonia, two patients died of systemic state changes, one patient died of gastrointestinal bleeding, and one patient died of bacterial septicemia with progressive renal failure. One patient with heart disease died 10 days after rituximab infusion. The patient mortality rates of 9.0% in the rituximab group, 1.9% in the omalizumab group, and 0.0% in the dupilumab group were all lower than the 11%-41% mortality rates of BP patients reported in other reports (3). The mean age of patients who died in the rituximab group was 59.3 years, including a 0.6-year-old child with BP, which led to this low figure. The mean age of patients who died after excluding this patient was 65.2 years, approximately equal to the mean age of patients in the rituximab group of 65.9 years. The age of patients who died in the omalizumab group was 78.0 years, higher than the mean age of patients in the omalizumab group of 68.2 years. The cause of death may be related to the patient’s advanced age and medical condition. However, the possibility that it was related to treatment with biologics cannot be ruled out, especially in the case of the one patient who died 10 days after rituximab treatment. However, the patient had a previous history of heart disease. Rituximab is currently the earliest and most widely used biological agent in treating BP. However, considering the incidence of adverse events and mortality, omalizumab or dupilumab may be more advantageous in treating BP.

A total of four children were included in the systematic review, of which three achieved complete remission after receiving rituximab, and one achieved partial remission after receiving rituximab. There were no adverse events but one patient died of respiratory failure three months after the last injection of rituximab. BP in children is very rare. The number of reported cases is less than 100 cases (101). The first-line treatment of BP in children is systemic corticosteroids but children are highly vulnerable to the side effects of systemic corticosteroids, notably infections and growth retardation (102). Rituximab is expected to replace corticosteroids as a more effective treatment for children with BP with fewer side effects, though more research data is needed. Some scholars believe that rituximab may lead to infection, drug fever, and other adverse reactions which should be used when a certain dose of corticosteroid is still unable to control the condition of the child. At present, there are no reports about the application of omalizumab and dupilumab in the treatment of BP in children. However, omalizumab has been widely used in the treatment of allergic asthma and urticaria in children and dupilumab has been widely used in the treatment of atopic dermatitis in children.

Limitations of this review include a small sample size and a lack of a control group. In addition, publication bias represents another limitation since studies with negative results are less likely to be published. Data reported in this systematic review are subject to publication bias of the underlying included studies consisting of case series and case reports (76.0%, n=57/75), retrospective (16.0%, n = 12/75), and small prospective studies (8.0%, n = 6/75). In addition, BP severity may affect rituximab, omalizumab, and dupilumab efficacy. However, the absence of this data and inconsistency of scoring methods in most of the included studies prevented further analysis.

## Conclusion

This systematic review comprehensively summarized the reports of rituximab, omalizumab, and dupilumab for BP treatment to date. Our data suggest that rituximab, omalizumab, and dupilumab have similar clinical benefits in BP. However, rituximab resulted in higher recurrence rates, adverse events, and mortality rates. Future randomized clinical trials are required to conclude the safety and efficacy of biologics in patients with BP.

## Data Availability Statement

The original contributions presented in the study are included in the article/supplementary material. Further inquiries can be directed to the corresponding author.

## Author Contributions

PC and WX: conception and design of the work, data collection, data analysis and interpretation, drafting the article. LZ: conception and design of the work, data collection, data analysis and interpretation, article revision and approval of the publication. All authors contributed to the article and approved the submitted version.

## Conflict of Interest

The authors declare that the research was conducted in the absence of any commercial or financial relationships that could be construed as a potential conflict of interest.

## Publisher’s Note

All claims expressed in this article are solely those of the authors and do not necessarily represent those of their affiliated organizations, or those of the publisher, the editors and the reviewers. Any product that may be evaluated in this article, or claim that may be made by its manufacturer, is not guaranteed or endorsed by the publisher.
